# Editorial: Acute pancreatitis infection: epidemiology, prevention, clinical characteristics, treatment, and prediction, volume II

**DOI:** 10.3389/fcimb.2026.1902222

**Published:** 2026-07-01

**Authors:** Wandong Hong, Maddalena Zippi, Mateusz Jagielski, Peter C. Ambe, Sirio Fiorino

**Affiliations:** 1 Department of Gastroenterology and Hepatology, The First Affiliated Hospital of Wenzhou Medical University, Wenzhou, Zhejiang, China; 2 Unit of Gastroenterology and Digestive Endoscopy, Sandro Pertini Hospital, Rome, Italy; 3 Department of General, Gastroenterological and Oncological Surgery, Nicolaus Copernicus University, Toruń, Poland; 4 Department of Surgery, Witten/Herdecke University, Witten, Germany; 5 Medicine Department, Internal Medicine Unit, Budrio Hospital Azienda USL, Bologna, Italy

**Keywords:** acute pancreatitis, infected pancreatic necrosis, infection, prediction, risk factor, severe acute pancreatitis

## 1 Introduction

Acute pancreatitis (AP) is a leading gastrointestinal cause of hospitalization worldwide and imposes a substantial healthcare burden (Huang et al., 2025). Although most cases are mild and self-limiting, approximately 20–30% progress to severe acute pancreatitis (SAP), characterized by persistent organ failure and high mortality (International Association of Pancreatology, 2025). Early identification of predictors for severe disease is therefore critical to selecting patients who would benefit most from intensified surveillance or early intervention (Hong et al., 2019). This Research Topic highlights recent advances in risk assessment for the severity and complications of acute pancreatitis, spanning predictive modeling, novel biomarkers, and long-term metabolic sequelae.

## 2 Severity of disease

Numerous clinical scoring systems, including the Ranson score and the Bedside Index of Severity in Acute Pancreatitis (BISAP), have been developed to predict disease severity, yet their accuracy remains only moderate (Hong et al., 2019). To improve predictive performance, Cao et al. enrolled 1,289 patients and developed ten machine learning models to predict SAP. The Light Gradient Boosting Machine (LightGBM) performed best, identifying serum calcium, white blood cell count, alpha-hydroxybutyrate dehydrogenase, and glucose as key predictors. The authors then built a Streamlit web tool for real-time risk assessment. When interpreting these findings, readers should note that the 2012 Atlanta criteria classify severity as mild, moderate, or severe (Banks et al., 2013); however, this study employed a binary classification of SAP versus mild acute pancreatitis (MAP), leaving the handling of moderately severe cases unclear. Furthermore, the model’s performance has not been validated on an external cohort.

Beyond predictive modeling, recent research has increasingly focused on biomarkers rooted in the pathophysiology of severe disease. Neutrophils, the most abundant innate immune cells, combat bacterial infections through phagocytosis, degranulation, and the formation of neutrophil extracellular traps (NETs) (Zhou et al., 2022). In AP, neutrophils are rapidly recruited to the inflamed pancreas, where increased NET formation exacerbates pancreatic injury. These activated neutrophils can also migrate retrograde into the circulation, contributing to systemic inflammation and distant organ damage (Wan et al., 2020). Consequently, NETs are key mediators of tissue injury in SAP (Hu et al., 2020). Given this central pathogenic role, the neutrophil-to-lymphocyte ratio (NLR) has been proposed as a useful predictor of SAP (Yang et al., 2015). Chooklin and Chuklin performed a systematic review and meta-analysis of 72 studies to define the role of NLR for primary risk stratification. They found that NLR measured within the first two days of hospitalization provided time-dependent prognostic information: at admission, NLR offered good discrimination for mortality, whereas its performance for discriminating severe disease and infection-related outcomes was moderate. Complementing this work, Hao et al. conducted a brief literature review summarizing emerging biomarkers, including serum markers such as serum amyloid A (SAA) protein, NETs, and extracellular vesicles, as well as intestinal flora and genetic markers such as mitochondrial DNA (mtDNA).

Another neutrophil-related biomarker is the CD64 antigen, a high-affinity IgG Fc receptor constitutively expressed on antigen-presenting cells such as monocytes but upregulated on neutrophils only upon activation, enabling clear discrimination between resting and activated states (Hoffmann, 2011). The neutrophil CD64 index, a flow cytometry-based ratio calculated as (MFI CD64 on neutrophils/MFI on lymphocytes) divided by (MFI on monocytes/MFI on neutrophils), reflects this upregulation and serves as an established diagnostic marker for acute inflammation and infection (Farah et al., 2017; Zhang et al., 2026). Shao et al. enrolled 302 patients and demonstrated that a higher neutrophil CD64 index (cut-off > 1.45) was associated with increased incidence of complications, including systemic inflammatory response syndrome (SIRS), acute respiratory distress syndrome (ARDS), and multiple organ failure. Thus, the neutrophil CD64 index may serve as a useful biomarker for severity risk stratification in acute pancreatitis.

## 3 Systemic complications

The systemic inflammatory response in SAP can lead to life-threatening organ dysfunction. Among these complications, acute respiratory distress syndrome (ARDS) is a severe form of respiratory failure caused by inflammatory pulmonary edema and hypoxia; outcomes remain hindered by a lack of effective predictive and preventive strategies (Shah and Rana, 2020). Within this Research Topic, Zhu et al. enrolled 351 SAP patients with ARDS (in-hospital mortality, 23.6%) and found that the lactate-to-albumin ratio within 24 hours of admission was independently associated with mortality, achieving an area under the receiver operating characteristic curve (AUC) of 0.70.

Another severe systemic complication, acute kidney injury (AKI), occurs in up to 70% of patients with SAP and significantly elevates mortality risk (Wajda et al., 2019). Current diagnosis relies on dynamic increases in serum creatinine and/or decreased urine output; however, a substantial rise in serum creatinine is a late indicator, prompting a continuous search for earlier biomarkers that could enable intervention before irreversible changes occur (Wajda et al., 2019; Wu et al., 2025). Addressing this challenge, Chen et al. developed a nomogram to predict AKI using data from 754 AP patients in the MIMIC-IV database and externally validated the model in 202 ICU patients at their local hospital, achieving an AUC of 0.628 in the external validation set.

## 4 Local complications

Beyond systemic involvement, approximately 20% of patients develop local complications such as pancreatic necrosis (Francken et al., 2025). In about one-third of these cases, the necrotic tissue becomes secondarily infected, typically during weeks 2 to 4 of the illness (Hamesch et al., 2025). Infected necrosis drives late clinical deterioration, often leading to persistent or new-onset organ failure with a mortality rate of up to 40% (Wolbrink et al., 2020). Its pathogenesis is linked to bacterial translocation from the gut, facilitated by disturbed intestinal motility, small bowel bacterial overgrowth, and increased mucosal permeability due to hypoperfusion (van Grinsven et al., 2016). Reflecting this understanding, treatment has evolved from aggressive surgery to a minimally invasive step-up strategy incorporating endoscopic internal drainage, lavage, and necrosectomy when required (Mahapatra and Garg, 2022). Within this Research Topic, Abulfaraj enrolled 119 patients in a Saudi tertiary center, of whom 21 (17.6%) developed infected necrotizing pancreatitis, and compared clinical features, microbial patterns, and outcomes between patients with and without infection.

A potential risk factor of secondary infection is persistent pancreatic enzyme elevation (PPEE)—defined as persistently high serum amylase or lipase beyond day 5 of admission—which may reflect impaired pancreatic duct clearance and juice stasis, conditions that favor bacterial translocation. Investigating this association, Lázár et al. conducted a prospective, multicenter cohort analysis of 912 patients with pancreatic fluid collections (276 with PPEE, 503 with normal enzyme kinetics [NEK]). Superinfection occurred more frequently in the PPEE group (25.0% vs. 16.0%; OR 1.86), and PPEE was also associated with a greater need for endoscopic ultrasound-guided and percutaneous interventions.

While most pancreatic necrotic collections resolve spontaneously with supportive care (Hui et al., 2019), intraperitoneal rupture and hemorrhage represent rare but devastating complications. Proposed mechanisms include enzymatic digestion of adjacent structures by activated pancreatic enzymes, local ischemia from organ compression, and portal or splenic vein compression leading to portal hypertension with subsequent hemorrhage (Hui et al., 2019). Timely risk assessment is essential for appropriate allocation of critical care resources. Addressing this need, Li et al. analyzed 181 patients with necrotizing pancreatitis and developed a nomogram incorporating body temperature, total protein, white blood cell count, and fibrinogen degradation products to predict intraperitoneal rupture, achieving an AUC of 0.897.

## 5 Pancreatogenic diabetes mellitus

Beyond the acute phase, AP can have lasting metabolic consequences. Approximately 23% of patients develop diabetes within three years of hospital discharge (Hart et al., 2021). This sequela, termed pancreatogenic diabetes mellitus (DM) or type 3c diabetes mellitus (T3cDM), has a multifactorial pathophysiology that remains incompletely understood (Olesen et al., 2022). Potential contributing factors include AP-induced autoimmunity, loss of islet cell mass, and endocrine functional impairment with insulin resistance (Hart et al., 2021). The degree of endocrine dysfunction may correlate with the severity of the acute attack, while obesity and hyperlipidemia may further increase the risk of functional impairment (Wu et al., 2011). As insulin resistance develops, a compensatory increase in insulin production may ensue, leading to elevated fasting plasma insulin levels and, in some cases, progression to hyperinsulinemia.

Advancing the clinical characterization of this complication, two studies in this Research Topic examined metabolic outcomes following acute pancreatitis. Qian et al. enrolled 388 patients and followed them for three years, identifying the largest amplitude of glycemic excursions (LAGE), a glucose variability index, as a useful parameter for risk stratification of post-acute pancreatitis diabetes mellitus. Focusing on a specific etiology, Wang et al. studied 92 patients with hypertriglyceridemia-induced acute pancreatitis and found that the hyperinsulinemia group had a higher frequency of ICU admission and a higher rate of readmission due to pancreatitis recurrence within 30 days after discharge, compared with the non-hyperinsulinemia group.

## 6 Conclusion

Although advances in supportive care and aggressive fluid resuscitation have reduced overall morbidity and mortality (Hart et al., 2021), early risk stratification of disease severity and its complications using machine learning models or single laboratory markers remains suboptimal. These markers either lack sufficient sensitivity and specificity or are not feasible for use in emergency departments of primary hospitals (International Association of Pancreatology, 2025). Future prospective, large-scale, multicenter clinical studies are needed to identify reliable biomarkers. Furthermore, the mechanisms and risk factors influencing intermediate- and long-term outcomes, such as pancreatogenic diabetes mellitus, remain poorly characterized (Hart et al., 2021). Elucidating the mechanisms of endocrine dysfunction during disease progression may open new therapeutic avenues for this debilitating sequela of acute pancreatitis (Hamesch et al., 2025).
